# A Novel Nanocomposite Membrane Combining BN Nanosheets and GO for Effective Removal of Antibiotic in Water

**DOI:** 10.3390/nano9030386

**Published:** 2019-03-06

**Authors:** Guohai Yang, Daqing Zhang, Cheng Wang, Hong Liu, Lulu Qu, Haitao Li

**Affiliations:** 1 School of Chemistry and Material Science, Jiangsu Normal University, Xuzhou 221116, China; 15605227012@163.com (D.Z.); 18852121622@163.com (C.W.); 2 Key Laboratory of Gas and Fire Control for Coal Mines (Ministry of education), China University of Mining and Technology, Xuzhou 221116, China; liuhong2013@cumt.edu.cn

**Keywords:** graphene oxide, boron nitride nanosheets, high permeance and selectivity, filtration membrane

## Abstract

Residual antibiotics in water have become a primary source of water pollution due to their misuse. Recently, membranes, produced by layered nanomaterials such as graphene oxide (GO), boron nitride (BN) and transition metal dichalcogenides, have been used in water purification, desalination and molecule separation as they are energy saving and simple to operate. The performance of membranes is closely related to their structure and the properties of the nanomaterials used. In this work, BN nanosheets (BNNSs) and GO were used to fabricate a two-dimensional nanocomposite membrane in order to improve the membrane’s permeance. It should be mentioned that the corresponding equal mass of the pure GO membrane was almost impermeable for the antibiotic solution. Multi-walled carbon nanotubes (MWCNTs) were inserted into the GO layers to increase the interlayer spacing and adsorb more antibiotics from the water. The resultant MWCNTs/BNNSs/GO membranes showed improved permeance and stable sieving capability for the antibiotic and small species. Specifically, permeance reached 30.2 L m^−2^ h^−1^ bar^−1^, which was much higher than pure GO membrane and the antibiotic rejection was 96.1%.

## 1. Introduction

Antibiotics, which are widely utilized in medicine, poultry farming and food processing [1,2], have attracted considerable attention due to their abuse and their harmful effects on human health and the ecological environment [3,4]. The misuse of antibiotics induces DNA contamination and accelerates the generation of drug-resistant bacteria and super-bacteria [5,6,7]; thus, some diseases are more difficult to cure [8]. A number of studies have revealed that the level of antibiotics in the soil, air and surface water, and even in potable water, is excessive in many areas [9,10,11], which will ultimately accumulate in the human body via drinking water and then damage the body’s nervous system, kidneys and blood system. Therefore, it is necessary to develop an efficient method to remove antibiotics present in water.

Membrane technology, which is characterized by being energy-saving, highly efficient and easy to operate, has been widely used in the last few decades. It has been applied in water desalination and purification, waste water treatment, gas separation, dialysis and so on. However, it is not easy to fabricate a robust membrane with high performance, high permeability, and high selectivity. Membrane performance is highly dependent on the materials used and their structure.

Recently, two-dimensional (2D) nanomaterials, such as graphene oxide (GO) [12,13,14], boron nitride (BN) [15,16], transition metal dichalcogenides [17,18,19], have been investigated in the membrane process due to their particular physical and chemical properties. For example, GO is appropriate for fabricating filter membranes due to its atomic thin thickness, lamellar structure and consequent specific properties, such as high chemical stability and specific surface, and its porous and rich oxygen-containing functional sites [20]. Moreover, GO-based membranes, using tunable interlayer spacing to intercept ions and contaminants in water, are most promising [13,21,22,23]. However, GO membranes do have some deficiencies. A number of methods and experiments have been performed to overcome the weaknesses of the original GO membrane, such as swelling or delamination in aqueous solutions and the tradeoff between selectivity and permeability [24]. A 2D heterostructure GO/g-C_3_N_4_@TiO_2_ membranes for water-oil separation with a permeance of 4536 L m^−2^ h^−1^ bar^−1^ has been fabricated [25]. Although permeation of the GO/g-C_3_N_4_@TiO_2_ membrane is very high, the performance of the membrane in removing contaminants such as antibiotics and dyes is unknown. Thiourea (TU) was used as a crosslinker to connect adjacent GO layers and fabricate a graphene oxide framework (GOF) membrane for the separation of small molecules [26]. The prepared TU-GOF membrane has good sieving properties for small molecules, resulting in almost complete rejection of alcohol and ions in the gas, solvent, and saline separations; however, the permeance of the membrane is low. Therefore, the fabrication of an efficient membrane to eliminate antibiotics from water is essential.

BN, another 2D nanomaterial which is known as “white graphene”, has unique properties distinct from graphene. Its characteristics include strong oxidation resistance at high temperatures, excellent adsorption capability, no surface charge and special luminescence. BN nanosheets (BNNSs) are remarkable substrates for graphene, MoS_2_ layers and other 2D nanomaterials in electronic and optical applications. Recently, BN has been widely used in membrane research area [27,28,29,30].

In this study, BNNSs and GO were combined to construct a 2D nanocomposite and form a membrane using vacuum filtration. BNNSs act as both substrate and filter in the membrane and replace integrant GO nanosheets which could prevent the swelling of GO membrane in aqueous solution. The fabrication of BNNS is easier and more energy-saving than that of GO, so the prepared membrane is likely to be produced on a large scale. Moreover, multi-walled carbon nanotubes (MWCNTs), decorated with polydiallyldimethylammonium chloride (PDDA), were inserted into the BNNSs/GO to increase the interlayer space and enhance the absorbability of the membrane. The prepared MWCNTs/BNNSs/GO nanocomposite membrane was characterized and then used to eliminate the antibiotic tetracycline hydrochloride (TCH) in water. Ultraviolet-visible (UV/Vis) spectrophotometry was utilized to detect the concentrations of TCH solution. The results show that the prepared membrane efficiently removed TCH in water with a quite high permeance of 30.2 L m^−2^ h^−1^ bar^−1^.

## 2. Experimental Section

### 2.1. Materials

Concentrated sulfuric acid (H_2_SO_4_, 98%), phosphorus penta-oxide (P_2_O_5_), potassium persulfate (K_2_S_2_O_8_), potassium nitrate (KNO_3_), potassium permanganate (KMnO_4_), hydrogen peroxide (H_2_O_2_, aq), hydrochloric acid (HCl), polydiallyldimethylammonium chloride (PDDA, aq, 20 wt%), hexagonal boron nitride (h-BN), TCH, and ethyl alcohol (EtOH) were purchased from Aladdin (Shanghai, China), graphite and MWCNTs (diameter 20–30 nm, length 10–30 μm) were purchased from BoYu Material Company (Beijing, China), deionized water was purified using the Milli-Q reference system (>18 MΩ·cm^−1^, Billerica, MA, USA). All chemicals were used directly without further preparation.

### 2.2. Fabrication of Graphene Oxide (GO) Dispersion

GO was fabricated using the modified Hummers’ method [31]. A mixed solution of concentrated H_2_SO_4_ (60 mL), K_2_S_2_O_8_ (4.2 g) and P_2_O_5_(4.2 g) was heated to 80 °C, and graphite powder (5 g) was added under continuous stirring for 4.5 h. When the solution had cooled, the products obtained were filtered and washed with deionized water until no residual acid was present. The dried fluffy product (5 g) was then placed into concentrated H_2_SO_4_ (120 mL) at 0 °C, followed by the controlled addition of KNO_3_ (2.5 g) and KMnO_4_ (16 g) with the temperature below 10 °C. The reaction was performed at 35 °C for 2 h, and continued for a further 2 h following the addition of deionized water (250 mL). Subsequently, deionized water (600 mL) and H_2_O_2_ (30 mL, 30 wt%) were added to the solution, and allowed to stand overnight. Finally, the mixture was washed with 1 M HCl solution and deionized water to confirm the removal of metal ions and acid, then dried at 60 °C for 2 days. The GO suspension at the concentration of 2 mg mL^−1^ was obtained after sonication. The GO suspension used in this study was all 2 mg mL^−1^.

### 2.3. Fabrication of Polydiallyldimethylammonium Chloride-Multi-Walled Carbon Nanotubes (PDDA-MWCNTs) Dispersion

PDDA solution (50 mg, 20 wt%) was diluted with 60 mL of deionized water and sonicated for 10 min, mixed with 10 mL MWCNTs (1 mg mL^−1^) solution and sonicated for another 30 min, and then centrifuged for 30 min at 5000 rpm. The supernatant was collected and filtrated to obtain PDDA-MWCNTs. The PDDA-MWCNTs was rinsed with 60 mL deionized water and dried at 60 °C overnight. The mass of PDDA-MWCNTs was weighed accurately, then the PDDA-MWCNTs was dispersed in water by sonication. Finally, a homogeneous PDDA-MWCNTs dispersion at a concentration of 0.5 mg mL^−1^ was prepared, and the PDDA-MWCNTs solution used below was all 0.5 mg mL^−1^.

### 2.4. Fabrication of Boron Nitride Nanosheets (BNNSs) Dispersion

The BNNSs dispersion was prepared using commercial h-BN powder by ball milling [32] (planetary ball mill, QM-3SP2). Typically, the h-BN powder (0.5 g) and urea (30 g) were added to a ball mill cell and ball milled at 500 rpm for 12 h at room temperature. During the high-energy ball-milling process, BN and urea particles were thoroughly mixed, and some small urea molecules may intercalate into the BN structure from the edges of BN layers. Powered by the energy of colliding balls, urea would decompose and lead to chemical bonding of NH_2_ group to few-layer BN.

After ball milling, the product was washed with deionized water to remove urea. The collected BNNSs was dispersed in water by sonication, then the dispersion was centrifuged for 30 min at 2000 rpm. The supernatant was collected and filtrated to obtain BNNSs. Finally, BNNSs dispersion at a concentration of 2 mg mL^−1^ was prepared by dispersing solid BNNSs in water. The dispersion was then characterized by zeta potential, scanning electron microscopy (SEM), transmission electron microscopy (TEM) and atomic force microscopy (AFM). The BNNSs dispersion used below was all 2 mg mL^−1^.

### 2.5. Fabrication of the Membrane

Six mL of PDDA-MWCNTs dispersion was added into 2 mL of GO dispersion and stirring for 10 min, then 4 mL of BNNSs dispersion was dropped into mixed solution under stirring. The final mixed solution was stirred at room temperature for 10 min, followed by sonication for 10 min, repeated 5 times. Then the mixed dispersion was used to fabricate the MWCNTs/BNNSs/GO membranes on a PVDF (polyvinylidene fluoride) membrane through vacuum filtration. The prepared membrane was used to filter the TCH solution and was characterized by SEM, TEM and X-ray diffraction (XRD). The total mass of the composite membrane was maintained at 15 mg. Specifically, the mass of PDDA-MWCNTs was constant at 3 mg and total mass of GO and BNNSs was constant at 12 mg. The mass ratio of GO and BNNSs was changed to 5:1, 2:1, 1:1, 1:2, and 1:5 to optimize the performance of the membrane. Moreover, the corresponding pure GO membrane, pure BNNSs membrane and MWCNTs/GO membrane were also produced to detect their performance for the removal of TCH from water. Specifically, 2 mL of GO dispersion was used to fabricate pure GO membrane, and 4 mL of BNNSs dispersion was used to fabricate pure BNNSs membrane. The MWCNTs/GO membrane was produced by 2 mL of GO dispersion and 6 mL of PDDA-MWCNTs dispersion.

### 2.6. Filtration Experiment

Five MWCNTs/BNNSs/GO membranes of equal weight (15 mg) were prepared under the same conditions, but the mass ratio of GO to BNNSs in the membrane was changed. The prepared membranes were used to filter 30 mg L^−1^ TCH solution by vacuum filtration and the vacuum degree was stabilized at 0.9 bar. The volume of TCH solution was 10 mL. The filter liquor was collected and detected by ultraviolet-visible (UV-Vis) spectroscopy. The rejection to TCH using the prepared membrane was calculated by the following formula: *η* = (*C*_0_ − *C*) * *C*_0_*^−^*^1^ * 100% = (*A*_0_ − *A*) * *A*_0_^−1^ * 100%, where *η* is the rejection of the membrane, *C*_0_ and *C* are concentration of TCH solution before and after filtration, *A*_0_ and *A* are maximal absorbance of TCH before and after filtration. The permeance of the membrane was calculated by the following formula: *J* = *V* * *S*^−1^ * *T*^−1^ * *P*^−1^. Where *J* is the permeance of the membrane, *V* is the volume of TCH solution, *S* is the area of the membrane, *T* is the filtration time and *P* is the vacuum degree. Moreover, different concentrations (0.2–0.8 mol L^−1^) of NaCl and CaCl_2_ solution were prepared to measure the effect of salts to the membrane performance. It is noted that the collected data were averages of three parallel tests. That is, three membranes were prepared for each condition at the same time.

### 2.7. Characterization

A scanning electron microscope (SEM) (Hitachi, Japan) and atomic force microscope (AFM) (Bruker, Germany) were used to observe the surface morphology and the thickness of the samples. A transmission electron microscope (TEM) (JEOL, Japan) was used to obtain the structures of the samples. X-ray diffraction (XRD) patterns (Shimadzu, Japan) of the samples were obtained to confirm the crystallographic properties, employing CuKα radiation, λ = 0.15418 nm. The zeta potential was measured with a SurPASS Electrokinetic Analyzer (Austria) with a clamping cell at 300 mbar. The UV-Vis spectrum (Thermo Fisher Scientific, Waltham, MA, USA) was used to monitor the concentration of the antibiotic.

## 3. Results and Discussion

### 3.1. Characterization and Mechanism

**Figure 1:**
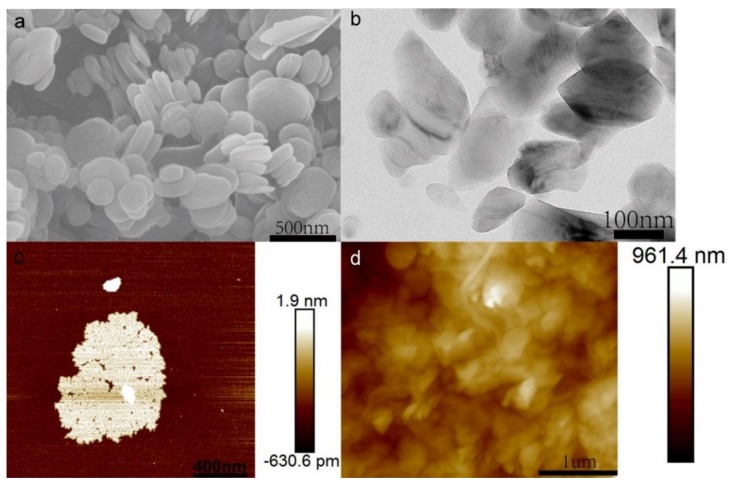
(**a**) Scanning electron micrograph (SEM), (**b**) transmission electron micrograph (TEM), and (**c**) atomic force micrograph (AFM) of boron nitride (BN) nanosheets. (**d**) AFM of the composite membrane.

The BNNSs used in this work were characterized by SEM, TEM and AFM, and the morphology images are shown in Figure 1. In Figure 1a, delaminated BNNSs were wafer-like and piled up randomly. The diameter of the BNNSs ranged from 100 to 500 nm which was consistent with the TEM and AFM results (Figure 1b,c). Figure S1a shows that the thickness of BNNSs collected by AFM was about 1.5 nm. These indicated that the BNNSs were successfully stripped from h-BN. The zeta potential of BNNSs dispersed in water was −36 ± 3 mV which is similar with the literature [32]. Figure 1d shows the surface morphology of the composite membrane. It can be seen that the surface of the membrane was rough due to the presence of BNNSs and MWCNTs. Moreover, the rough surface helped to retain the contaminant in water. The prepared GO was also characterized by SEM, TEM, and AFM (Figure S1b–d), which demonstrated the successful synthesis of single-layer GO.

**Figure 2:**
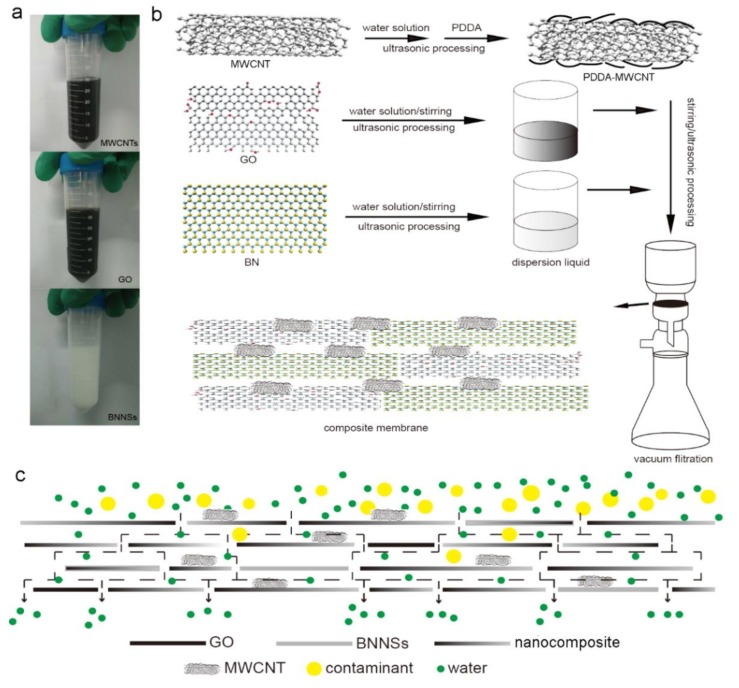
(**a**) A photograph of the prepared multi-walled carbon nanotubes (MWCNTs), graphene oxide (GO) and boron nitride nanosheets (BNNSs) dispersion. (**b**) Preparation flow chat of the membrane. (**c**) Illustration of the solute separation mechanism.

The dispersion liquids of BNNSs, GO and modified MWCNTS, shown in Figure 2a, were used to build the skeleton of the composite membrane using vacuum filtration and the preparation process is shown in Figure 2b. After the modification with PDDA, a cationic polyelectrolyte, the positively charged MWCNTs dispersion was mixed with the negatively charged GO dispersion and stirred for 10 min, then the negatively charged BN dispersion was dropped into the mixing system under stirring. In order to mix the dispersions evenly, the mixed liquid was stirred for 10 min and sonicated for 10 min (repeated five times) before vacuum filtration. GO and BNNSs were stacked on each other, and subsequently built the skeleton of the membrane which was the water transportation channel.

**Figure 3:**
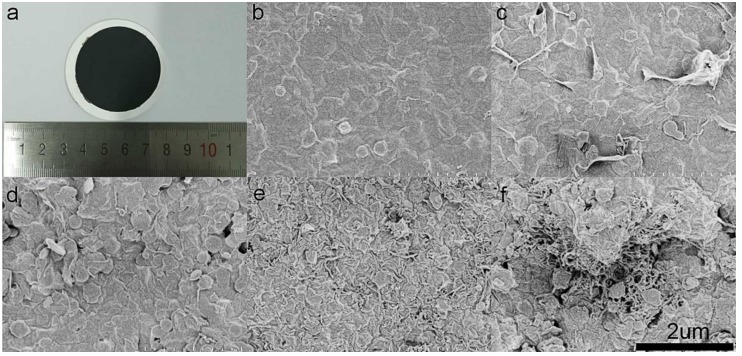
(**a**) A photograph of the prepared nanocomposite membrane (**b**–**f**) SEM images of the Membrane-X, X = 5, 2, 1, 0.5, 0.2, respectively. The scalebar of (**b**–**f**) is 2 μm.

**Figure 4:**
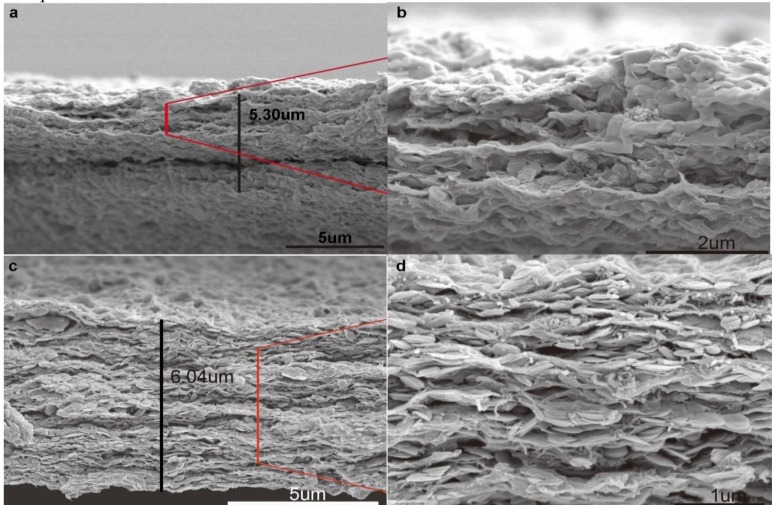
Cross section of the prepared membrane with different volume dispersion. (**a**,**b**) 6 mL dispersion. (**c**,**d**) 12 mL dispersion.

An optical image of the MWCNTs/BNNSs/GO membrane is shown in Figure 3a and a commercial hydrophobic membrane was used as the substrate. It should be mentioned that the membrane was flexible and stable in water. In order to maximize the permeance of the membrane and obtain high contaminant molecule trapping, the mass ratio of GO to BNNSs was changed. The mass ratio of GO to BNNSs was 5:1, 2:1, 1:1, 1:2, and 1:5, respectively, and the corresponding membrane was denoted as Membrane-X (M-X) where X is the mass ratio of GO to BNNSs (X = 5, 2, 1, 0.5, 0.2). As observed in Figure 3b–f, the surface of the membrane became rough when the mass of GO was decreased. The membrane skeleton which formed the nanochannel was replaced by the BNNSs/GO nanocomposite. Thus, the larger interlayer space of the membrane led to a greater permeance. At the same time, the MWCNTs in the membrane helped to adsorb contaminants due to their nanostructure. The effect of MWCNTs on membrane performance was also determined (Figure S2). It was found that 3 mg MWCNTs was optimal for membrane performance. The thickness of the composite membrane was simply adjusted by filtering different amounts of mixed dispersion (Figure 4). As shown in the image, the thickness of the membrane fabricated using 12 mL of mixed dispersion was 6.64 μm, while the membrane fabricated using 6 mL of mixed dispersion was 5.30 μm. It is possible that the skeleton of the thin membrane was looser. The membrane used in subsequent studies was fabricated using 12 mL of mixed dispersion.

**Figure 5:**
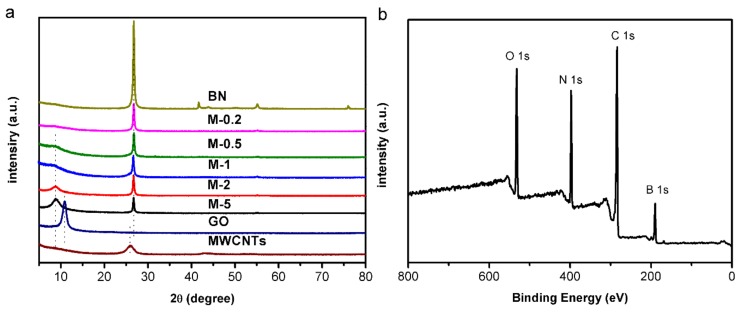
(**a**) X-ray diffraction (XRD) pattern of Membrane-X and the materials used. (**b**) X-ray photoelectron spectroscopy (XPS) spectra of the prepared membrane.

Figure 5a shows the XRD patterns of the prepared MWCNTs/BNNSs/GO membrane with different mass ratios of GO to BN. Pure GO and BN membranes were also characterized. The characteristic diffraction peak of pure GO membrane appeared at 10.9°, and the characteristic diffraction peak of the prepared composite membrane appeared at 8.79° for GO (001) and 26.8° for BN (002), indicating that the interlayer space of GO increased from 0.81 nm to 1.01 nm. The reason for the increased GO interlayer space is that BNNSs and MWCNTs embed in adjacent GO sheets. The interlayer space of BN remained unchanged. The characteristic diffraction peak of MWCNTs was not observable in the membrane. This may due to the fact that MWCNTs were surrounded by GO and BNNSs. When the mass ratio of GO to BN changed, the peak of GO gradually became invisible, in accordance with the SEM results, which may be attributed to the replacement of GO by the BNNSs/GO. In addition, Figure 5b shows the X-ray photoelectron spectroscopy (XPS) survey scan spectra of the prepared membrane, specifying the co-existence of C, N, O, and B elements. The B 1s peak at 189.8 eV is accredited to BNNSs in the membrane, and it indicated that the BNNSs/GO nanocomposite membrane was formed.

The possible contaminants removal process is illustrated in Figure 2c. Due to the similarity between GO and BNNSs in terms of lattice structure, GO and BNNSs easily formed membrane via facile vacuum filtration [33] (Figure 2b). The synergistic effect of the membrane’s size exclusion and MWCNTs’ adsorption contributed to the successful contaminants’ removal. Specifically, the BNNSs/GO skeleton made up the nanosized channel, which prevented the passage of macromolecular contaminants and allowed water flow at the same time. Moreover, the intercalation of MWCNTs increased the interlayer space of the membrane, thus resulting in a faster water flow and the MWCNTs enhanced the adsorption of contaminants. It is also suggested that electrostatic interactions between functional groups on the BNNSs/GO nanosheets and the charged contaminants helped to retain the contaminants [34]. Due to the use of BNNSs, the interlaminar water transportation channel became anfractuous, which was beneficial for the removal of contaminants. However, the permeance of the membrane did not decrease due to the increase in interlayer space and the smaller lateral size of BNNSs (100–500 nm) than that of GO (500–1000 nm). With the increasement of BNNSs, the joint point of nanomaterial increased in the layer, leading to the formation of through-thickness cracks which allow filtrate easily across the layer. Therefore, the prepared membrane was able to maintain high permeance and result in high rejection.

### 3.2. Membrane Performance

**Figure 6:**
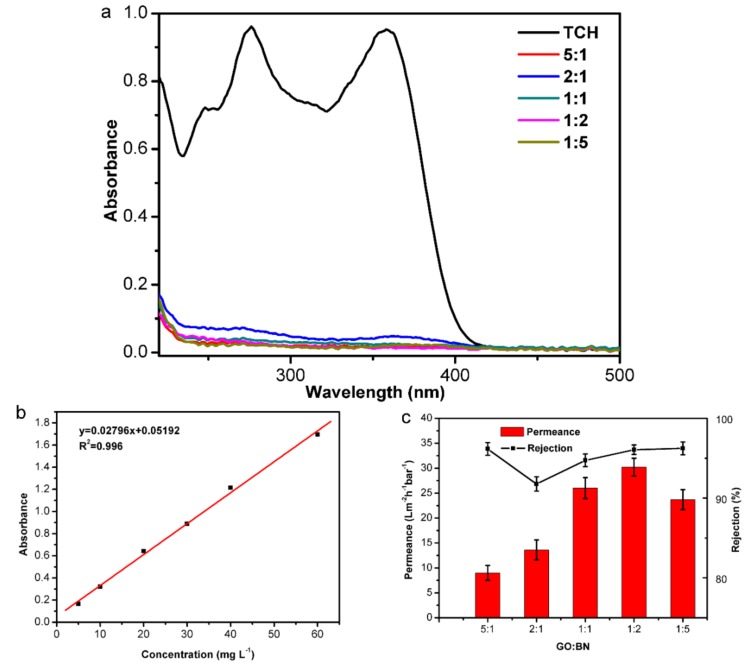
(**a**) Ultraviolet-visible (UV-Vis) absorption spectra of initial tetracycline hydrochloride (TCH) (concentration of 30 mg L^−1^) and the filtrate through the M-X. (**b**) standard curve of the concentration of TCH (5, 10, 20, 30, 40, 60 mg L^−1^). (**c**) Filtration performance of Membrane-X.

The UV-Vis absorption spectra of TCH solution (30 mg L^−1^, 10 mL) before and after filtration using the composite membranes with different GO and BNNSs mass ratios are shown in Figure 6a. Figure 6b is the standard curve which shows the relationship between maximal absorbance (261 nm) and concentration of TCH solution. It was obvious that the absorbance of TCH solution after filtration sharply declined which indicated the effective removal of TCH. By absorbance, the rejection to TCH was easily obtained. Moreover, the permeance of the composite membrane was also monitored (Figure 6c). When considering both the rejection to TCH and the permeance, it was concluded that the best performance of the composite membrane was achieved when the mass ratio of GO to BNNSs was 1:2 as the rejection reached 96.1% which was higher or similar to that in other reports [35,36]. In addition, the permeance was higher than in other reports [37,38,39,40] and was 30.2 L m^−2^ h^−1^ bar^−1^. Possible reasons for the increase in permeance are as follows: With an increase in BNNSs, the number of BNNSs/GO nanocomposites increased, and then BNNSs/GO replaced GO as the membrane skeleton. The permeance increased due to the larger interlayer space of BNNSs/GO. However, when the number of BNNSs was too high, BNNSs replaced the BNNSs/GO, resulting in a decrease in the interlayer space and permeance.

**Figure 7:**
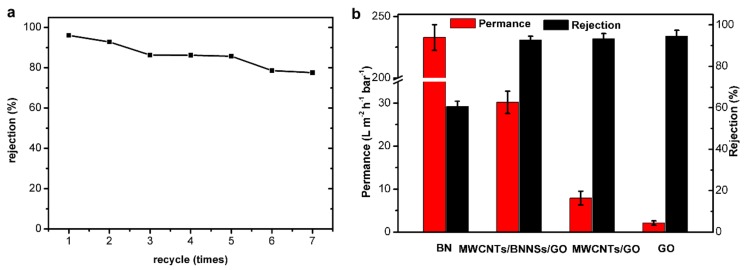
(**a**) Rejection of the prepared membrane to TCH during 7 times cycle (10 mL, 30 mg L^−1^ TCH solution was used for each time). (**b**) Performance of BN, MWCNTs/BNNSs/GO, MWCNTs/GO, GO membrane for removal of TCH.

When the volume of TCH increased to 20 mL, the membrane rejection to TCH was still high and reached 92.8% (Figure S3). On this basis, a further experiment was performed to test the recycling ability of the membrane. As shown in Figure 7a, the rejection rates of the membrane for TCH was still above 75% after a cycle of 7 times. This is different from simple adsorption whose rejection to contaminants has a sudden drop [41]. This also demonstrated the good stability of the prepared MWCNTs/BNNSs/GO membrane for the removal of TCH from water. The performance of MWCNTs/BNNSs/GO membrane was compared with pure BN, GO and MWCNTs/GO membrane, and the result was shown in Figure 7b. The permeance of pure BN membrane was as large as 232.9 L m^−2^ h^−1^ bar^−1^, but the rejection to TCH was only 60.1%. In contrast, the pure GO membrane and MWCNTs/GO membrane showed good rejection to TCH which were above 93%, but their permeances were slow (below 8 L m^−2^ h^−1^ bar^−1^). The prepared MWCNTs/BNNSs/GO membrane showed both high rejection of TCH and relatively large permeance. Furthermore, the rejection of PVDF polymer substrate to TCH was also explored, but it showed almost no rejection to TCH. Therefore, the membrane’s size exclusion is the main reason for the high rejection.

**Figure 8:**
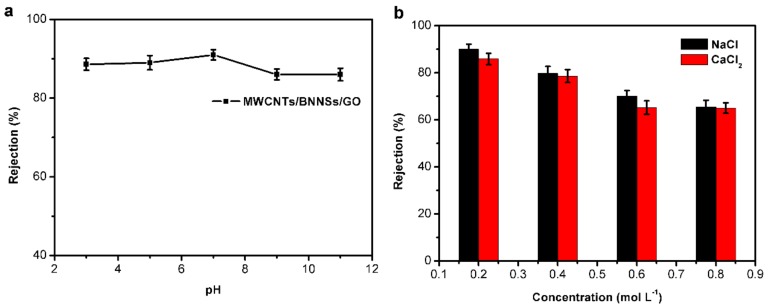
(**a**) Effect of pH to the membrane performance. (**b**) Effect of saline solution on the membrane performance.

In order to determine the performance of the membrane in environments such as acidic, alkaline or saline conditions, a series of experiments were performed by adjusting the pH of the TCH solution or adding salt into it. Figure 8a shows the influence of pH value on the rejection of TCH, and it was found that the performance of the membrane declined slightly in alkaline solution compared with acid or neutral solutions. A possible reason for this is that the hydrochloric in TCH dissociated and reacted with the alkali, then producing salt which made the environment harsher and reduced membrane performance. Although the rejection in alkaline condition declined, it still reached 86.1%. Figure 8b shows the effect of different salt concentrations on the membrane performance. It can be seen that the higher the salt concentrations, the lower the membrane performance. This result demonstrated that salts induce a harsher environment when the membrane intercepts TCH from solution. As the color of the TCH solution changed due to the addition of alkaline or salt, reactions may have occurred in the solution. The color of TCH solution was clear and transparent after filtration. It is very likely that electrostatic interactions occurred between metal cations and negatively charged BNNSs, and these electrostatic interactions partly destroyed the interaction between GO and BNNSs, resulting in a decline in membrane performance. However, the membrane was still stable in a salts solution and had a blocking effect on saline ions.

## 4. Conclusions

In summary, a novel BNNSs/GO nanocomposite membrane was developed using a facile method, with a suitable performance for removing antibiotics in water. MWCNTs were used as a filler to increase the interlayer space of the membrane and absorb contaminant molecule. The membrane showed a steady rejection to TCH and a high permeance. Moreover, the membrane was stable in harsh environments such as acidic, alkaline and saline, and was still effective. When the amount of contaminant was increased, the membrane performance was still considerable. The combination of a simple, easy operation preparation process and excellent performance makes this membrane a promising option in water treatment.

## Supplementary Materials

The following are available online at https://www.mdpi.com/2079-4991/9/3/386/s1, Figure S1: (a) AFM of the BNNSs, (b) SEM, (c) TEM, and (d) AFM of GO nanosheets; Figure S2: Filtration performance of membrane with different weight of MWCNTs, from group 1 to 5, the weight of MWCNTs was 0.5, 1, 2, 3, 4 mg, respectively; Figure S3: (a) UV-Vis absorption spectra of the initial TCH solution and filtrate, the filtrate obtained by filtrating 20 mL 30 mg L^−1^ TCH using the prepared membrane. (b) Filtration performance of membrane with different mass ratio of GO to BN after filtrating 20 mL 30 mg L^−1^ TCH.
