# Supplementary material for: A Novel Nanocomposite Membrane Combining BN Nanosheets and GO for Effective Removal of Antibiotic in Water
Source: Nanomaterials (Basel). 2019 Mar 6;9(3):386. doi: 10.3390/nano9030386 (PMC6473978; doi:10.3390/nano9030386)
Supplement: Supplementary file 1 [file nanomaterials-09-00386-s001.pdf]

Supporting Information

A Novel Nanocomposite Membrane Combining BN nanosheets and GO for Effective Removal of Antibiotic in Water

Guohai Yang[*#1], Daqing Zhang[#1], Cheng Wang[1], Hong Liu[2], Lulu Qu[*1], Haitao Li [*1]

1.School of Chemistry and Material Science, Jiangsu Normal University, Xuzhou 221116, China.

2. Key Laboratory of Gas and Fire Control for Coal Mines (Ministry of education), China University of Mining and Technology, Xuzhou 221116, China.

*To whom correspondence should be addressed:

E-mail: yangguohai@jsnu.edu.cn; luluqu@jsnu.edu.cn; haitao@jsnu.edu.cn

[#] These authors contributed equally to this work.

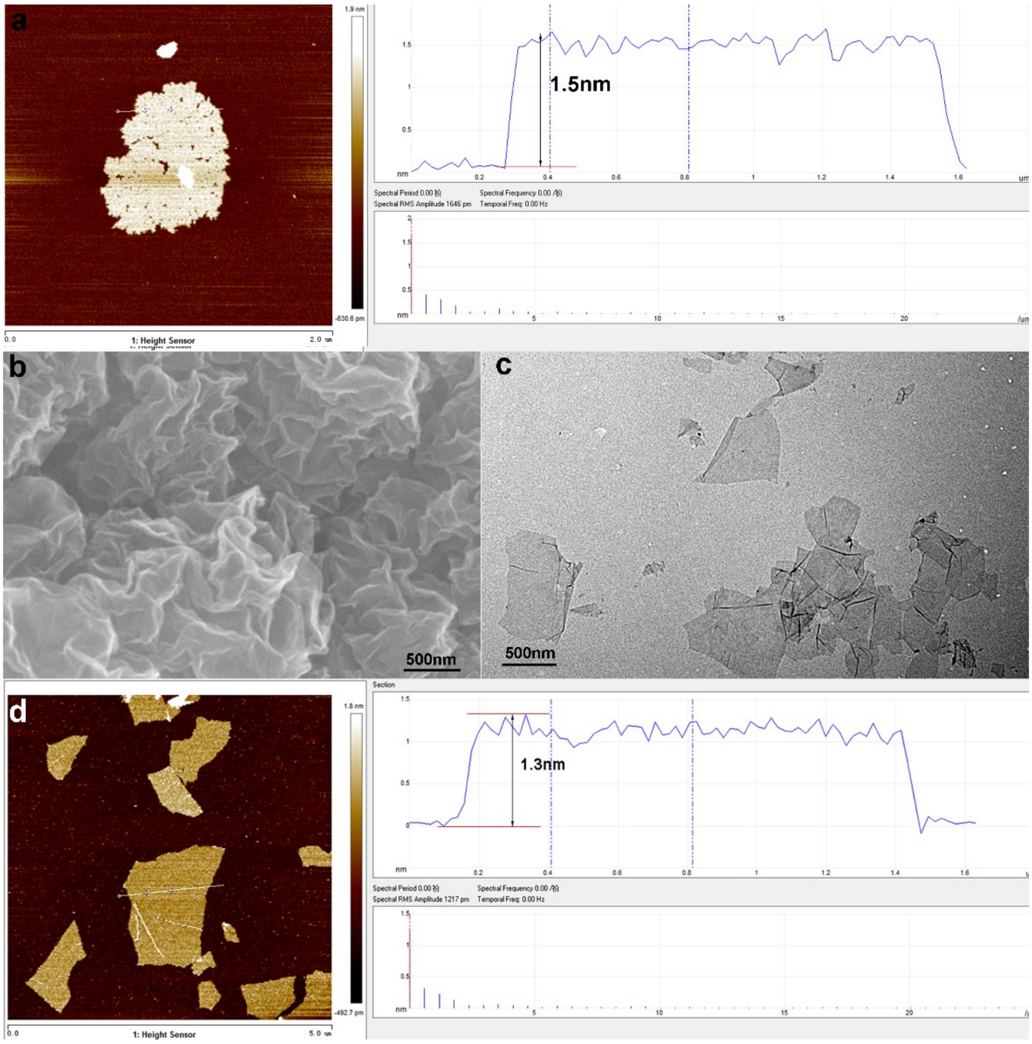

Fig. S1 (a) AFM of the BNNSs, (b) SEM, (c) TEM, and (d) AFM of GO nanosheets.

AMF was used to measure the thickness of BNNSs, and the result (Fig S1a) showed that the thickness of BNNSs was about 1.5 nm. The GO nanosheets were fabricated through the modified Hummers' method. The size of the prepared GO were about 500-1000 nm and the morphology were shown in Fig. S1b, c, d.

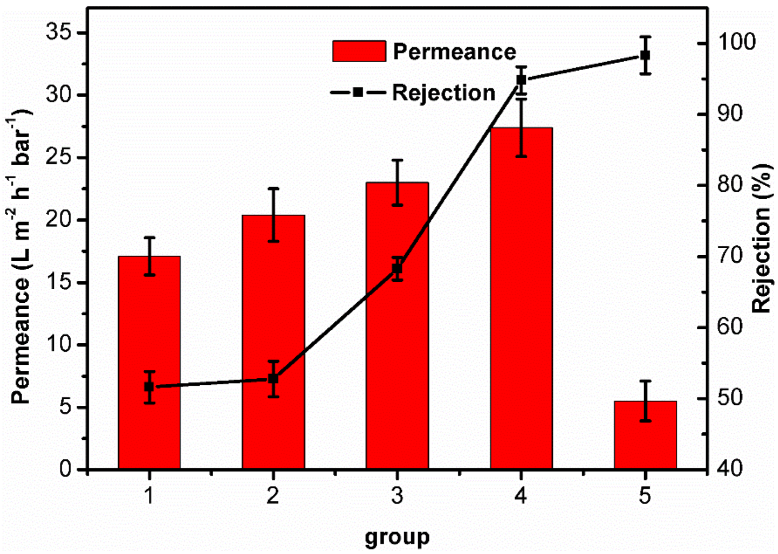

Fig. S2 Filtration performance of membrane with different weight of MWCNTs, from group 1 to 5, the weight of MWCNTs was 0.5, 1, 2, 3, 4 mg, respectively.

The mass of the MWCNTs in the membrane was confirmed by experiments. The mass ratio of GO to BN was fixed at 1:1, then the mass of MWCNTs was changed. From Fig. S2, we confirm the mass of MWCNTs at 3 mg where the membrane performance is integrated.

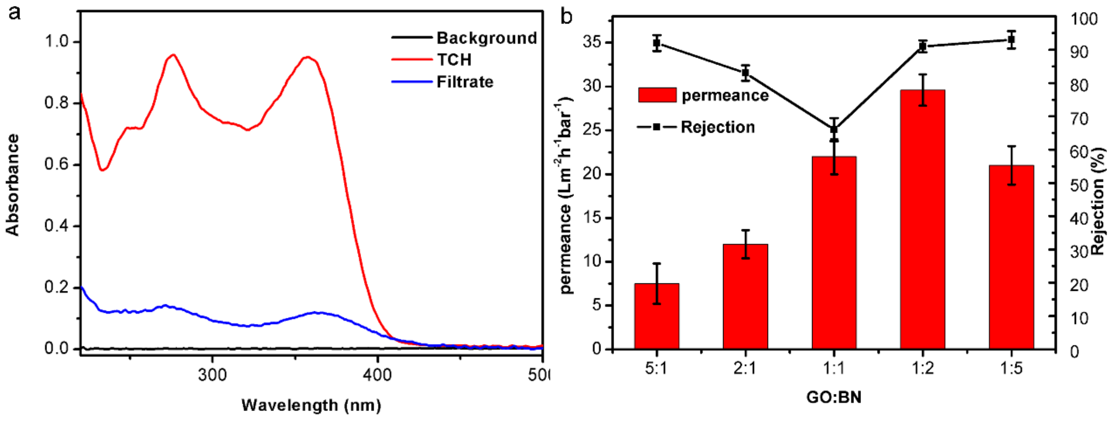

Fig. S3 (a) UV-Vis absorption spectra of the initial TCH solution and filtrate, the filtrate obtained by filtrating 20 mL 30 mg L^{-1} TCH using the prepared membrane. (b) Filtration performance of membrane with different mass ratio of GO to BN after filtrating 20 mL 30 mg L^{-1} TCH.
